# Development of a nurse home visitation intervention for intimate partner violence

**DOI:** 10.1186/1472-6963-12-50

**Published:** 2012-02-29

**Authors:** Susan M Jack, Marilyn Ford-Gilboe, C Nadine Wathen, Danielle M Davidov, Diane B McNaughton, Jeffrey H Coben, David L Olds, Harriet L MacMillan

**Affiliations:** 1 School of Nursing, McMaster University, 1280 Main Street West, Hamilton, ON, Canada; 2 Arthur Labatt Family School of Nursing, The University of Western Ontario, London, ON, Canada; 3 Faculty of Information and Media Studies, The University of Western Ontario, London, ON, Canada; 4 Department of Emergency Medicine, West Virginia University, Morgantown, WV, USA; 5 College of Nursing, Rush University, Chicago, IL, USA; 6 Departments of Emergency Medicine and Community Medicine, West Virginia University, Morgantown, WV, USA; 7 Prevention Research Center for Family and Child Health, University of Colorado Denver, Denver, CO, USA; 8 Offord Centre for Child Studies, McMaster University, Hamilton, ON, Canada; 9 West Virginia University Injury Control Research Center, Morgantown, WV, USA

## Abstract

**Background:**

Despite an increase in knowledge about the epidemiology of intimate partner violence (IPV), much less is known about interventions to reduce IPV and its associated impairment. One program that holds promise in preventing IPV and improving outcomes for women exposed to violence is the Nurse-Family Partnership (NFP), an evidence-based nurse home visitation program for socially disadvantaged first-time mothers. The present study developed an intervention model and modification process to address IPV within the context of the NFP. This included determining the extent to which the NFP curriculum addressed the needs of women at risk for IPV or its recurrence, along with client, nurse and broader stakeholder perspectives on how best to help NFP clients cope with abusive relationships.

**Methods:**

Following a preliminary needs assessment, an exploratory multiple case study was conducted to identify the core components of the proposed IPV intervention. This included qualitative interviews with purposeful samples of NFP clients and community stakeholders, and focus groups with nurse home visitors recruited from four NFP sites. Conventional content analysis and constant comparison guided data coding and synthesis. A process for developing complex interventions was then implemented.

**Results:**

Based on data from 69 respondents, an IPV intervention was developed that focused on identifying and responding to IPV; assessing a client's level of safety risk associated with IPV; understanding the process of leaving and resolving an abusive relationship and system navigation. A need was identified for the intervention to include both universal elements of healthy relationships and those tailored to a woman's specific level of readiness to promote change within her life. A clinical pathway guides nurses through the intervention, with a set of facilitators and corresponding instructions for each component.

**Conclusions:**

NFP clients, nurses and stakeholders identified the need for modifications to the existing NFP program; this led to the development of an intervention that includes universal and targeted components to assist NFP nurses in addressing IPV with their clients. Plans for feasibility testing and evaluation of the effectiveness of the IPV intervention embedded within the NFP, and compared to NFP-only, are discussed.

## Background

Over the past two decades, there has been a substantial increase in what is known about the epidemiology of intimate partner violence (IPV). In a multi-country World Health Organization (WHO) study, the lifetime prevalence of physical or sexual violence ranged from 15 to 71% [1]. There is now a significant body of research that documents a broad range of mental and physical health problems associated with IPV [1-4]. Among pregnant women, past and current IPV has been associated with an increased risk of pregnancy and childbirth complications [5]. Despite greater recognition of IPV as a major public health problem [6] and evidence that women exposed to IPV are high users of health care services [7,8], much less effort has been given to developing interventions aimed at reducing IPV or its consequences compared with the emphasis on universal screening [9].

A number of systematic reviews [9-13] have concluded that the evidence supporting specific interventions for abused women is weak, especially interventions provided in health care settings, or those to which health care providers could refer women. A recent review [12] found some evidence that advocacy interventions (i.e. information and support to deal with abuse and to access needed services) can improve women's quality of life, safety actions, social support, access to services and lead to a reduction in violence, especially among those who have disclosed abuse or who seek help from shelters. Success varies by the type and intensity of the intervention. Coordination of services ("one-stop-shopping") and taking into account women's help-seeking strategies and abuse experiences may improve service effectiveness. However, the effectiveness of shelter services in reducing violence and improving other outcomes for women remains understudied.

The evidence for batterer treatment and for couple therapy is mixed, with the better-designed studies generally indicating no benefit (e.g., [14]), or potential harm (i.e., increased recidivism) [15,16]. Most authors caution that couples therapy is not safe for many abused women, particularly those in relationships where there is a high degree of coercive control, characteristic of intimate partner terrorism [17]. There is evidence that permanent, but not temporary, civil protection orders may be effective in reducing future violence [18].

There is emerging evidence regarding the positive impacts of specific types of counselling, case management or support interventions delivered by nurses [19,20] or social workers [21] in reducing IPV and improving other outcomes in specific groups: low income, urban, pregnant, African American women, [21] low income, urban, ethnically diverse, American women [19] and pregnant Chinese women accessing prenatal care in Hong Kong [20]. While results of these studies are promising, further evaluation of these interventions with larger, more diverse samples, and using rigorous methods, is warranted [19-21].

One program that holds promise as an approach to reducing IPV and improving outcomes for women exposed to violence is the Nurse-Family Partnership (NFP) [22]. An evidence-based nurse home visitation program for low income, first-time mothers, the NFP has been shown, in three randomized controlled trials (RCTs), to improve maternal and child health, including reduction of injuries and child maltreatment [23]. Results from the first NFP trial conducted in Elmira, New York showed that, in nurse-visited households where the mother reported moderate to severe levels of IPV, the beneficial program effect on child maltreatment was not found, [24] suggesting the need to develop more effective ways of helping women address IPV within the context of the NFP.

The NFP is an intensive program that begins prenatally and follows women until the child is two years of age. Addressing IPV was not an original goal of the program, yet the long-term relationship between the nurse and woman presents a potentially important opportunity for intervening with women who are at risk of IPV. Of note, in one NFP trial, nurse-visited women reported significantly less exposure to IPV in the previous six months at the four-year follow-up, compared with those in the control group, [25] suggesting that there may be practices embedded within the way the NFP is delivered that could be enhanced to improve program effectiveness among women experiencing IPV. Furthermore, both NFP nurses and their supervisors have identified that IPV is a challenging issue that is often encountered in their practices. These factors prompted the development of an intervention to address IPV within the context of the NFP.

Since the NFP was not designed specifically to respond to women exposed to IPV, the first step was to determine the extent to which the program provided education and support to assist nurses in addressing this issue. A web-based survey of NFP nurses and supervisors, conducted in 2006 as a preliminary study to this project, indicated that almost 40% felt they did not have sufficient knowledge and skills to adequately address IPV and approximately 72% reported that IPV in the home made delivering the NFP somewhat or very difficult. At the time, the NFP training curriculum included minimal information about assessing IPV, client safety and the effects of IPV on children.

The context of nurse home visitation provides a unique opportunity to deliver services to women exposed to IPV. The development of a therapeutic nurse-client relationship, built on a foundation of acceptance, trust and strong rapport is at the core of most long-term home visiting programs with vulnerable populations [26,27]. It is this relationship that may facilitate nurse home visitors' abilities to ask about IPV and increase clients' comfort levels in disclosing IPV exposure [28]. Working with families in their home also creates opportunities to assess the quality of interpersonal relationships and the potential to identify IPV before it begins or escalates [29].

The purpose of the present study was to develop an IPV intervention to embed within the NFP that would be evaluated in a subsequent trial to determine its effectiveness in reducing IPV and improving quality of life. This intervention addresses *intimate partner terrorism*, a particularly serious type of IPV characterized by physical violence, combined with coercive control (i.e. threats, economic control, use of privilege and punishment, using the children, isolation, emotional abuse and sexual control), and almost always directed by a man toward a female partner [17].

In this paper, we summarize the process that was conducted to develop the IPV intervention and provide an overview of the intervention's primary elements.

## Methods

**Figure 1:**
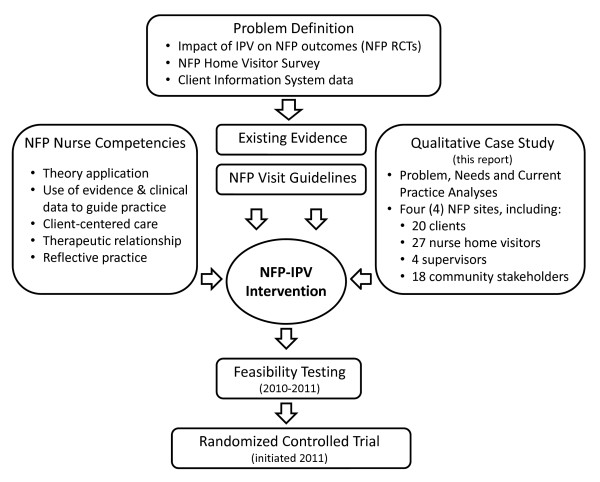
**Development of the intervention to address IPV in Nurse-Family Partnership home visitation program**.

We adapted van Meijel and colleagues' [30] model for developing complex, evidence-based nursing interventions which includes: 1) defining the problem; 2) identifying the necessary "building blocks" for the design; 3) developing the intervention; and 4) piloting and evaluating the modified intervention in a clinical trial (Figure 1). This model outlines a framework for collecting and using qualitative data in the development stage that we used to ensure that the resulting intervention is tailored to the specific educational and practice needs of nurses delivering health promotion and illness prevention services in the home setting. This attention to context, in particular understanding and responding directly to the needs of socially disadvantaged first-time mothers, and taking into consideration the collaborative partnerships that exist between the NFP agencies and other community partners, is vital in maximizing the potential effectiveness of a new or adapted intervention [22,31].

To identify the intervention "building blocks" or core components, an exploratory multiple case study [32] was conducted to: 1) understand the problem of IPV as it is experienced by women enrolled in the NFP program and described by NFP nurse home visitors and nurse supervisors; 2) identify women's needs and requests for intervention through the NFP program; 3) analyze the current practices nurses use to identify and address the needs of women exposed to IPV; and 4) analyze the strategies and organizational policies that exist to support women exposed to IPV in the community. Permission to conduct this study was obtained from the Hamilton Health Sciences/McMaster Faculty of Health Sciences Research Ethics Board, and the Institutional Review Boards of West Virginia University and the University of Colorado. The research protocol was also reviewed and approved at the NFP National Service Office (NSO) and at each local NFP site by the appropriate committee or an IRB within an affiliated academic center.

### Site selection

US NFP sites were informed through electronic communication from the NFP NSO about the study to develop an IPV intervention. Eight sites volunteered to participate and from this group, four sites were selected based on the following inclusion criteria: 1) site has graduated at least one cohort of clients; 2) nurses are providing services to women exposed to IPV; and 3) no current involvement in other research studies. The selection of four NFP sites for this study facilitated the process of identifying similarities and differences both within and between cases [33]. With the support of the NFP NSO, we met with site administrators to explain the study objectives and methods. To assist us in identifying common themes emerging across diverse contexts, we employed maximum variation sampling to select sites that varied by rural/urban or urban context and length of time that a site had been in operation. Using this type of purposeful sampling strategy allowed for the identification of shared common experiences of the phenomena, core concepts and central themes that "cut across cases and derive their significance from having emerged out of heterogeneity." [[34], p. 235]

### Samples and data collection

As triangulation of data sources is a hallmark of case study research [32], purposeful samples of NFP clients, nurse home visitors and community stakeholders were invited to participate in the study so that we could develop an in-depth, multi-perspective understanding of the issue of responding to IPV within this home visitation program. Prior to data collection, we obtained informed consent from each study participant, and permission to audiotape all interviews and focus groups. Each individual also completed a short demographic questionnaire. At the end of each interview or focus group, we completed comprehensive field notes to document observations, key themes emerging from the interview, and questions for further follow-up.

#### NFP client interviews

From among the clients enrolled in each of the participating NFP sites, a purposeful sample of pregnant women and mothers who met the following inclusion criteria were invited to participate in the study: 1) they had reported exposure to IPV within the past year to their nurse home visitor or on the modified Abuse Assessment Screen [35], a self-report questionnaire routinely administered to women within the first three visits of the NFP program; 2) were 16 years or older and; 3) able to converse in English. To achieve data saturation with this fairly homogenous sample, we estimated recruiting 20 women in total (approximately five from each site). Data saturation is the point in time in the study when it is determined that informational redundancy has been achieved and that further interviewing will not result in the emergence of new core themes [34]. Each participant was invited to complete two face-to-face, in-depth interviews conducted by local research assistants who had professional or volunteer experience working with vulnerable women and children.

Each interview was conducted in a private room within a public building (e.g. library, health department). The length of the interviews ranged from 30-90 minutes. We followed a training and safety protocol for interviewing women exposed to IPV, based on that used by members of this research team in a previous multi-site trial assessing IPV screening [36]. Women received a $30 gift card to acknowledge their time in completing each interview.

The focus of the first interview was to understand the mothers' perspectives regarding: violence in her lifetime intimate relationships, decisions about disclosure of IPV exposure to her nurse home visitor, and the nature and acceptability of supports received from her nurse and other sources. In the second interview, the research assistant shared a brief summary of the first interview to determine accuracy of the research team's interpretation of the woman's account, followed by open-ended questions designed to elicit mothers' perceptions of strategies that could be implemented in the NFP to identify IPV, and reflections about how IPV exposure had affected her health and that of her child. Additionally, women's perceptions about the acceptability of two assessment tools: 1) the Domestic Violence Survivor Assessment (DVSA) [37] and; 2) the Composite Abuse Scale (CAS) [38] were explored. Each client was also asked to read five scenarios developed to depict situations of IPV exposure similar to what study participants may have been exposed to in their own relationships, and then to reflect on how a NFP nurse home visitor could best support the woman depicted in the narrative. The five scenarios were designed to represent varied points in the process of disengaging from an abusive relationship, modelled after those used on the DVSA [37]: 1) committed to continuing the relationship; 2) committed to, but questioning aspects of the relationship; 3) views herself as abused and begins to consider her options; 4) breaking away and; 5) establishing a new life. We anticipated that asking mothers to reflect on these scenarios would de-personalize the experience and be a less threatening method for exploring this sensitive issue [39], while still providing an opportunity to speak about their own experiences if they wished to do so.

#### NFP nurse interviews

Agencies that implement the NFP program contract to adhere to 18 program model elements to ensure fidelity to the intervention model evaluated in the RCTs. As part of this model, agencies are required to hire registered nurses who hold a minimum of a baccalaureate nursing degree (although certifications may slightly vary across agencies due to different state regulations). In the NFP program, all full-time nurse home visitors are required to maintain a caseload that does not exceed 25 clients.

All of the nurse home visitors at each of the four NFP sites were invited to participate in two focus groups, six months apart, for a total of eight focus groups. The purpose of the focus groups was to extend the problem analysis, including identification of potential barriers to implementing the current NFP curriculum with women exposed to IPV. As part of the practice analysis, nursing strategies currently used to resolve client issues related to IPV and to meet client identified needs were explored. At each site, a senior member of the research team (SJ or HM) facilitated the two focus groups; a doctoral student (DD) participated in the co-facilitation of four groups.

We developed semi-structured interview guides for each focus group with NFP nurses. The first focus group explored: a) reflections on NFP mothers' experiences of the problem and their needs; 2) experiences in working with women and children exposed to IPV; 3) identification of current strategies used to identify, assess and respond to IPV; 4) recommendations for strategies to enhance management of IPV within the NFP program; and 5) client, organizational, cultural and systemic factors influencing nurses' abilities to respond to IPV.

The second set of focus groups began with an opportunity for the nurses to reflect upon and respond to summaries of nurse and client data collected up to that point. The new topics explored during these groups included: 1) the knowledge and skills required by nurses to address IPV during home visits; 2) issues regarding nurses' perceived competence and capacity to respond to IPV; 3) perceptions of the acceptability of the DVSA and the CAS; and 4) the feasibility of using motivational interviewing techniques [40] to enhance client self-efficacy and stimulate behavior change. The focus groups lasted between 120-150 minutes and permission to record the discussions was obtained.

#### Community stakeholder interviews

These interviews were conducted to identify facilitators and barriers to augmenting the NFP program to address IPV and to discuss the feasibility of implementing intervention components suggested by NFP mothers and nurses. A purposeful sample of community-based stakeholders involved in responding to IPV or delivering services to women and children exposed to violence was invited to participate in the study. To achieve saturation, we estimated recruiting approximately five stakeholders per site, for a total of 20 individuals. Snowball sampling was used to identify these individuals by asking NFP administrators to identify the individuals within their communities who could provide a rich description of the issues under exploration. NFP nurse supervisors were also invited to participate. Respondents were asked to complete two semi-structured interviews, each lasting approximately 60-90 minutes.

The purpose of the first interview was to understand the stakeholder's current role in supporting women exposed to IPV and to identify organizational, system and cultural influences on developing enhanced NFP interventions to support this population. At the beginning of the second interview, each stakeholder was provided with a summary of their initial interview and asked to comment on the accuracy of our interpretation. Building upon themes emerging across datasets, the second interview with each stakeholder explored issues related to: strategies for supervising professionals working with families exposed to violence; the role of their agency in collaborating with the NFP to address client needs; the role for nurse home visitors in intervening with perpetrators of violence; and their perceptions of the specific nurse home visitor role in responding to IPV. Each stakeholder was provided with a $25 gift card as an honorarium for participating in the study.

### Data analysis

We transcribed verbatim all recorded interviews with identifying information removed. Data analysis was conducted concurrently with data collection in order to identify themes requiring further exploration. NVivo 8.0 software was used to store, index and code all of the data. The principles of conventional content analysis [41] and constant comparison [42] guided all coding and synthesis of the data. Four team members (SJ, MFG, DM, DD) participated in coding and summarizing the data.

### Intervention development

Four data sources were used to develop the NFP IPV intervention (Figure 1). First, the results of the case study informed the content and structure of the intervention, as well as the processes to train nurses in intervention delivery, support supervisors, and engage clients in the work of addressing violence in their lives. Second, evidence drawn from existing quantitative and qualitative research informed the theoretical foundation of the intervention e.g. [17,43-45] and was used to augment assessment techniques [46], responses to IPV disclosures [47,48] and intervention procedures [20,49-53]. Theoretical principles of the NFP program and attention to NFP nurse competencies were also integrated into the intervention components. Finally, some content from the existing NFP visit guidelines was adapted and then integrated into the intervention.

The qualitative data emerging from the focus groups with the nurse home visitors and the interviews with NFP clients and community stakeholders were used to develop a training curriculum for nurse home visitors and an intervention that was specific to the home visitation context. Broadly the data were used to identify and define: 1) What are the primary problems encountered in identifying and addressing IPV within this context?; 2) What knowledge and skills do nurses and supervisors need to respond to the issue of IPV?; 3) What are clients' needs and their expectations of their nurse home visitors?; and 4) What current practice strategies are either problematic or are a promising clinical strategy to include in the intervention.

Although data were collected across four unique NFP sites, similar core themes emerged across this diversity with regards to nurses' professional development; the types of IPV clients are exposed to, the challenges and opportunities to assess for IPV; how nurses respond to IPV disclosures; clients' expressed needs for safety planning, social support, and information about healthy relationships; and the availability and accessibility of community resources to support clients and nurses. Across the four NFP sites, differences were noted, specifically the awareness of different cultural norms or responses to violence among client populations and the impact of working with abused women in urban versus rural settings. In developing the intervention, attention to these differences was addressed in one of two ways: 1) the development of curriculum materials that a client and nurse could choose to use if it was relevant to the family's situation (e.g. a facilitator on safety planning in rural or farm communities); and 2) provision of resources to support nurses in tailoring the intervention to their clients' unique situations.

We worked from the premise that the IPV intervention needed to fit cohesively with the current NFP home visitation guidelines and to reflect the underlying philosophy of the NFP program. As the intervention is an enhancement to the current NFP curriculum, it was also essential to ensure that the intervention was developed on a framework of existing NFP nurse home visitor competencies which include: 1) applying theories and principles integral to the implementation of the NFP model [54]; 2) utilizing the best available evidence along with data from the NFP Efforts to Outcome (ETO™) system to guide and improve practice; 3) delivering individualized care across the six NFP home visit domains; 4) establishing a therapeutic relationship with the client; and 5) utilizing reflective processes to improve practice. To achieve these goals, the research team included the originator of the NFP (DO) and his colleagues from the Prevention Research Center. Additionally, two researchers (SJ, HM) participated in and completed the full NFP nurse home visitor and supervisor training and consulted extensively with nurse educators and nurse consultants from within the NFP NSO. All of the IPV intervention facilitators and nurse instructions were subsequently formatted in a manner consistent with current NFP guidelines.

## Results

We present here an overview of results from the qualitative case study - i.e., the focus groups with NFP nurses and the interviews with clients and community service providers and stakeholders - that directly influenced development of the IPV intervention. We then describe the complex intervention, including each of the five components, including guidelines, protocols and tools for implementing each, as well as methods used to monitor its implementation.

### Participant and site characteristics

Four NFP sites located in the Midwest region of the US participated in the qualitative case study (Table 1). The sites selected varied by the size of the agency (as determined by the total number of clients ever enrolled), regional population, and the geographical location of the population (urban or urban/rural mix). There was variability in the years of experience each agency had in implementing the NFP (range 2-12 years). Additionally, each NFP site was ethnically diverse in terms of the women enrolled in the program, with nurses visiting predominantly in: Site 1-white/non-Hispanic women (77.5%); Site 2 -white/non-Hispanic (35.7%) and black/African American (45.4%) women; Site 3- black/African American (67.2%) women; and Site 4- Hispanic (71%) women.

**Table 1:** Characteristics of NFP case study sites

	NFP Site 1	NFP Site 2	NFP Site 3	NFP Site 4
**U.S. region (Census Bureau designated area)**	Midwest (West North Central)	Midwest (East North Central)	Midwest (East North Central)	Midwest (East North Central)
**Population 2006 estimate **[55]	90,056	733,203	156,771	515,269
**Population served**	Rural/urban mix	Urban	Urban	Rural/urban mix
**Number of nurse**	5	8	7	7
**home visitors**				
**Year NFP initiated**	2000	2006	1996	2000
**Client characteristics of all NFP clients enrolled at site**				
**Total number clients ever**	630	185	1653	637
enrolled**				
**Median age**	20	18	17	17
**High school completion rate**	68.5%	38.4%	26.6%	20%
**% unmarried**	85%	95.7%	97.8%	92%
**% Medicaid recipients**	58.3%	70.3%	63.3%	90%
**Race/Ethnicity**				
White/non-Hispanic	77.5%	35.7%	28.6%	14.0%
Hispanic	5.5%	4.3%	1.0%	71.0%
Black/African American	5.3%	45.4%	67.2%	12.0%
Multiracial/other	6.0%	11.9%	3.0%	3.0%
Asian/Pacific Islander	2.2%	1.1%	0.1%	
Native American	3.6%	1.6%	0.1%	

**As of September 30, 2008

All of the individual NFP clients, nurse home visitors, and community stakeholders who were invited to participate in the study, provided consent and completed an initial interview or focus group. Across the four sites, a total of 69 individuals shared their perceptions and experiences about the role of the NFP in identifying and addressing IPV. Twenty NFP clients with current or past exposure to IPV participated in the study. Of these 20 NFP clients (mean age = 21 years; mean years of school completed = 12); half of them indicated that they were currently in a relationship; 40% were currently single with no partner and 10% indicated that they were separated or divorced. Reflecting the complex lives of many young, low-income mothers, the participating NFP clients reported a range of living situations: 10% were homeless; 30% were living alone with their child(ren); 15% were living with a partner and 45% were living with extended family members. From the original 20 NFP clients, 16 participants were located and consented to complete the second interview (one client per site was lost to follow-up, despite multiple attempts to contact and locate them).

All of the nurse home visitors employed within these four sites, for a total of 27 nurses (mean age = 44 years) participated in at least one focus group; 22 of the 27 nurse home visitors participated in both of the focus groups conducted at their site. The reasons for not participating in both of the focus groups included: a) engagement in a prior commitment; b) inability to commute to the office on that particular date; or c) personal illness. The majority of the nurses (77%) were educated at the Bachelor degree (or higher) level with the remaining nurse home visitors holding an Associate Degree in Nursing (23%). The participating home visitors had extensive professional nursing experience (mean 20 years; range 5-38 years) and had been NFP nurse home visitors for an average of four years. Each nurse who participated confirmed that she had experience in assessing clients for their exposure to IPV or with working with an NFP client who had disclosed a history of current or past abuse. The small purposeful sample of nurses who participated in this study is fairly typical compared to the full population of nurse home visitors employed by the NFP across the US. In the US, the mean age of nurse home visitors is 45.4 years with 80.2% of nurses educated at the Bachelor level and 12.5% holding an Associate Degree in Nursing; overall NFP nurse home visitors have a mean of 14 years of nursing experience and a mean of 3.8 years of employment as a NFP home visitor [56].

A total of 22 community stakeholders, including the NFP nurse supervisors from each site, participated in the study and completed both interviews. The majority of these individuals had a graduate level education (77%) with the rest educated at Bachelor's level or equivalent (23%). These stakeholders represented agencies across diverse sectors including: domestic violence agencies (including shelters, advocacy services and crisis centers) (41%); justice or law enforcement (27%); health (23%) and education (5%).

### Client experiences and needs for an IPV intervention

For many of the NFP clients interviewed, exposure to violence was a common and expected experience. The women described experiencing multiple types of abuse in their lives including childhood maltreatment, witnessing the abuse of their own mothers, experiencing previous IPV or dating violence themselves, or living in communities where violence was prevalent. While some reported entering partner relationships to get away from an abusive home, others still lived with parents who were abusive. Women tended to have few economic resources, little support and perceived that their options for change were limited because they were "trapped" by structural issues including economic insecurity, unstable housing and a lack of access to quality day care. In contrast, those women who reported higher levels of support from family members were also more likely to have more stable living conditions, at home with their parents or on their own, and more options for responding to IPV. In spite of these challenges, most of the women interviewed had left their abusive partner or were attempting to leave or end the relationship.

The NFP clients highly valued the relationship with their nurse home visitor - a relationship, once established, that could facilitate IPV disclosure. Within this sample, most of the participants had disclosed their experiences of abuse to the nurse, either on their own or in response to being asked, but reported that there were risks associated with disclosure including: losing the nurses' respect, feeling shame and embarrassment, increased involvement of child protection services, fear that their partner would find out and the violence would increase, or that the nurse would not maintain confidentiality. Clients also expressed concern that they would experience a loss of control over decisions for themselves and their children if following a disclosure the nurse started to tell her "what to do." However, overall the clients indicated they would support being asked frequently, within the context of discussions about relationships, about their safety and exposure to past or current IPV.

In the needs analysis, the women identified seven broad categories of suggestions about how the NFP nurse could best support abused women, including: 1) actively listening, acknowledging, accepting and understanding women's experiences of abuse; 2) proactively asking about IPV and providing information about community resources and healthy relationships; 3) supporting the client to build her confidence and assisting her to set goals; 4) identifying and discussing options and choices available, without providing "advice;" 5) engaging women in safety planning; 6) assisting women to learn about and navigate health, social, education, child care, employment, justice, domestic violence, and housing services; and, 7) encouraging connections with other women to share strategies for dealing with violence and changing their lives.

From the participants' responses to the five scenarios depicting different stages of change, we identified topics to be prioritized within each stage of the intervention to be tailored to women's readiness to respond to violence, as outlined in Table 2.

**Table 2:** IPV-Specific Strategies by DVSA Stage

Stage of change re: IPV	Strategies endorsed by NFP clients
1. Committed to continuing the relationship	Nurse:
	• asks about safety
	• provides information about healthy relationships
	• provides information on local community resources

2. Committed to, but questioning, the relationship	Nurse provides information about:
	• the health and social impacts of IPV on women and
	children
	• the cycle of abuse
	• strategies for responding to abuse
	• community resources and processes for accessing
	support

3. Views herself as abused and begins to consider her options	Nurse:
	• helps woman explore the strengths and limitations of
	their relationships
	• provides options for addressing IPV
	• helps woman engage in safety planning

4. Breaking away	Nurse:
	• continues to focus on safety planning
	• helps woman access resources and navigate the system
	• "encourage and support" rather than "push"

5. Establishing a new life	Nurse:
	• continues to provide tailored support to help women stabilize and maintain their situation
	• facilitates access to formal and informal social supports

### Nurses' experiences of addressing IPV within home visiting practice

Participating NFP nurses explained that, although few clients in their individual caseloads were dealing with IPV at any given time, working with such women was very time and resource intensive. Nurses confirmed that the abuse experienced by their NFP clients included severe physical, emotional and financial abuse by highly controlling partners; women had been choked, shot with a gun or electroshock weapon, or received injuries that required medical attention or hospitalization. Some women fought back, heightening nurses' concerns about their safety.

The nurses confirmed that addressing a client's exposure to IPV was problematic for many reasons. Many clients lacked the resources needed to help them deal with the abuse including support from their families, and access to safe housing, social and domestic violence services and health care, including treatment for mental health and substance use problems. Nurses perceived these women to be very protective of their partners, to fear living and parenting alone and, yet consumed by attempts to cope with the abuse. In this context, the ability to engage women in the NFP and to deliver core program content was seen as a challenge.

Nurses also identified that, once trust was established, they typically learned about their clients' exposure to violence in two ways: 1) the client initiated disclosure, usually following a violent episode or a pattern of escalating violence; or 2) as a result of completing a relationship assessment that was regularly conducted by the nurse home visitor. Nurses expressed concern and frustration about not knowing how to facilitate an IPV disclosure when they had a "gut feeling" that a client was being abused. For clients who did disclose, many nurse home visitors described confidence in providing an immediate empathic response to the woman.

From the practice and needs analyses, it emerged that the nurse home visitors had specific knowledge and skill deficits in identifying and responding to IPV. It was apparent that nurses required a deeper understanding of the cycle of abuse, the types of violence women are exposed to within intimate relationships, clinical risk indicators suggestive of IPV exposure, the impact of IPV exposure on women's health and on children; and the trajectory or stages of abusive relationships. With respect to broader social and system level issues, the nurses identified the need to better understand how to deliver culturally-sensitive care to new immigrants and varied ethnic populations, and how justice, housing, and law enforcement services respond to the issue of violence.

Nurses also identified that they needed opportunities to further develop skills in: comprehensive assessment of IPV; engaging abused women in safety planning; using motivational interviewing to help women explore feelings of ambivalence about the abuse and to meet their stated goals; conducting risk assessments; and tailoring interventions to the client's level of readiness to address IPV and safety. Within the context of delivering the core NFP curriculum, nurses expressed needs to develop strategies for supporting mothers in their parenting role within the context of an abusive relationship, to understand and address their clients' violent behaviour towards their partners, to support women in navigating social service systems, and to work with women to help them define the perpetrators' behaviors as abusive. Additionally, from our analysis of current nursing practice, we identified that nurses require additional skills and supports in establishing therapeutic boundaries with women and children exposed to violence, how to retain clients following an IPV disclosure and how to manage feelings of compassion fatigue or frustration. To enhance their training, nurses expressed a preference for participating in interactive workshops and viewing videos highlighting how expert clinicians assess and engage with abused women. They also wanted written scripts, illustrating how to talk about IPV or how to introduce specific assessment tools or facilitators within a home visit.

### Stakeholders' perceptions: Problem and needs analysis

Professionals who partner with NFP nurses in addressing IPV within the community cited challenges in responding to IPV similar to those expressed by the nurses and clients. They also highlighted that many abused women have limited access to community services because of a lack of transportation, few financial resources and for some, limited proficiency in English. The stakeholders interviewed were supportive of NFP nurses increasing their involvement in identifying and intervening with abused women and children. Professionals from the domestic violence and justice sectors identified that their agencies could support NFP nurses in this role by providing: agency tours, ongoing information about how to navigate different services, training and skill development, and consultations about difficult cases.

### Intervention components

**Figure 2:**
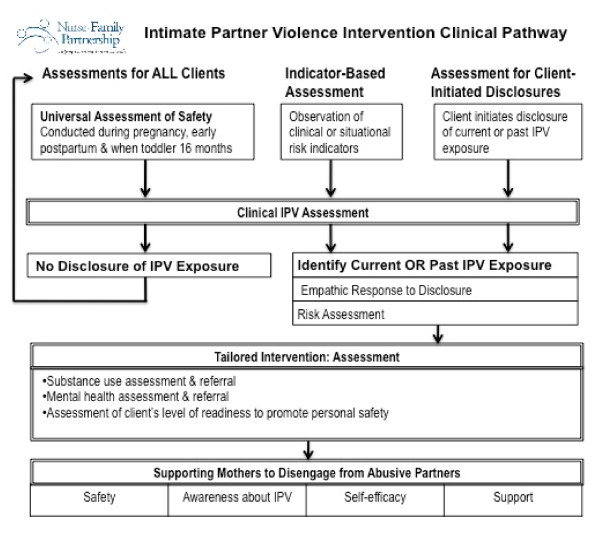
**NFP IPV Intervention Clinical Pathway**.

The data emerging from the qualitative case study, along with existing NFP curriculum materials and theoretical and empirical evidence from the literature, were used to develop a complex intervention [57] that includes five interconnecting components: 1) a curriculum to train nurse home visitors to identify and respond to IPV; 2) a manualized intervention including a clinical pathway (Figure 2); 3) guidelines for reflective supervision; 4) a site readiness checklist and; 5) ongoing coaching to support adoption and uptake of the intervention into home visiting practice.

### Nurse training curriculum

The training curriculum was developed to increase nurses' knowledge about IPV and their level of competence in identifying, assessing and intervening with women and children exposed to IPV. Findings from the qualitative data informed the development of the curriculum objectives, the type of content to be included, and the teaching-learning strategies to be implemented. The curriculum is comprised of five modules: 1) defining IPV; 2) identifying and responding to IPV in a home visit; 3) assessing a client's level of risk associated with IPV; 4) understanding the process of leaving and resolving an abusive relationship and; 5) system navigation. Multiple teaching and learning strategies were developed to support nurses in completing the training objectives. Individual level learning strategies included: independent study to understand the theoretical foundations and components of the intervention; watching training videos that include demonstrations of a nurse integrating the NFP IPV intervention into a home visit and a presentation on research foundations of the Danger Assessment instrument for intimate partner femicide [58] and the process for administering and scoring the tool; and completing online certification [59] to administer this risk assessment tool. Group learning strategies were also integrated into the curriculum, allowing the nurses opportunities in their regular team meetings to role play and practice using the intervention facilitators and materials; discuss strategies for integrating the intervention into home visiting practice; reflect on facilitators and barriers to working with NFP clients exposed to IPV; and meeting with community partners to learn about local community resources and services, including the process for obtaining protection orders. The training concludes with a one-day in person consolidation workshop led by a nurse-researcher (SJ, MFG) to review the process for delivering the NFP-IPV intervention.

#### Manualized intervention and clinical pathway

The NFP IPV intervention consists of both universal and targeted assessment opportunities and tailored intervention strategies based on a client's level of readiness to address abuse, and is intended to: 1) reduce the woman's exposure to IPV; 2) reduce the child's exposure to IPV; 3) improve the client's quality of life; 4) increase the number of safety strategies adopted by the client; 5) enhance maternal self-efficacy; 6) increase a woman's awareness about the impact of IPV; and 7) assist her in identifying and accessing informal and formal social supports. A clinical pathway (Figure 2) guides nurse home visitors through the intervention, with a set of facilitators and corresponding instructions for each pathway component, briefly described below.

#### Assessing IPV exposure

The *universal assessment of safety*, conducted at three points of time, is an opportunity to: 1) discuss the importance of safety as a basic human right; 2) explore maternal perceptions of feeling safe or unsafe in her community, home, and relationships; 3) gently introduce the concept of power and control in relationships; and 4) explore the client's perception of her intimate partner relationship. Exposure to IPV may also be identified by the nurse initiating an indicator-based assessment when she observes clinical or situational risk indicators associated with IPV or when the client initiates a disclosure during the course of a regular home visit.

#### Assessing IPV severity and immediate safety

Following the identification and disclosure phase, a clinical IPV assessment is conducted to confirm exposure and understand the nature, pattern and severity of abuse experienced by the woman. In most circumstances, a positive response to any of the questions on the clinical IPV assessment indicates that a client is experiencing abuse in her current relationship (or the past 12 months) and should receive all elements of the IPV intervention, which is delivered by nurses using non-judgmental, active listening to validate the woman's experiences and affirm that she is not to blame, while acknowledging the difficulty of living with abuse, respecting the client's autonomy in decision-making and ensuring confidentiality. As part of the immediate response, an assessment of the client's level of risk is conducted using the Danger Assessment [58], followed by a brief discussion of key safety strategies and, as necessary, initiating actions to address immediate safety concerns. Additionally, the nurse asks the client about, and explores the potential advantages and risks of, her options for responding to the violence. Written examples of scripts that could be adapted by nurses to introduce these tools into a home visit were developed.

#### Tailored assessment and intervention

After risk assessment and brief safety planning, the nurse explores the client's level of readiness to address the issues of violence in her relationship using five evidence-based narratives, developed from the qualitative client interviews, that describe different mothers' stories of survival and strength. The stories emphasize that making changes to improve safety is a process and there is no "right way" to do this- women must work through this process in a way that fits with their lives and stage of readiness. However, the wisdom shared by other women through the stories is a good starting point for considering options. By identifying the story to which the client most closely relates, the nurse can identify appropriate ways to tailor the intervention to meet her needs.

For some women, mental health and substance use issues are closely associated with their exposure to violence and trauma, and each of these consequences of IPV can make it more difficult for women to make changes in their lives to improve their safety. Therefore, as part of the IPV intervention, nurses screen clients for mental health and substance use concerns with tools already in use at the local site. If clinical concerns are detected, the nurse identifies appropriate community resources and, where possible, supports the woman to access local resources and/or treatment.

Based on the initial assessment of readiness to address IPV, the nurse tailors subsequent intervention elements in four areas: Safety (S), Awareness about IPV (A), Self-efficacy (S), and Support (S) (SASS). Educational hand-outs and activity worksheets, designed to structure and guide the nurse-client interaction, were developed for each area. In the clinical pathway, nurses are recommended to complete at least one of the SASS facilitators per home visit, based on the client's level of readiness and preference, along with the nurse's judgment. To accompany each educational hand-out, an instruction page for nurses was developed that included information on how to introduce the facilitator into a home visit, background information on the concepts to be discussed, sample open-ended questions to use to explore the issue with the client as well as information on additional resources.

### Guidelines for reflective supervision

One of the core elements of the NFP program model is that supervisors are required to provide nurses with regular opportunities for clinical supervision and reflection on their practice. As part of the NFP-IPV intervention, guidelines for clinical reflective supervision were developed for NFP supervisors to support nurse home visitors in understanding and resolving issues that arise in their work with clients exposed to current or past IPV. Examples of such issues include how to support nurses in maintaining appropriate therapeutic boundaries or how to address nurses' feelings of frustration when clients do not choose to leave an abusive partner.

### Site readiness checklist

A site readiness checklist was developed to ensure that NFP nurses have adequate support and structure to implement the IPV intervention. This checklist is reviewed by the supervisor prior to intervention implementation to ensure: a) the organization has a policy to address the safety of nurses during home visits; b) a plan exists for documenting IPV exposure in client records; c) all nurses are aware of the local reporting mandates for child and adult exposure to IPV; and, d) local procedures for conducting mental health and substance use assessments and referrals exist.

### Coaching to support intervention adoption and implementation

The final component of the NFP-IPV intervention involves coaching for intervention uptake and implementation to: provide ongoing review and reinforcement of the intervention's theoretical foundations and components; identify site-specific strategies for implementing intervention elements; and to problem-solve for specific issues. A clinical consultant, a registered nurse with content expertise about the IPV intervention and the NFP program, meets via teleconference with the implementing NFP nurse supervisor every four to six weeks, or as issues arise. The coaching sessions also provide the supervisor an opportunity for reflective supervision.

## Discussion

We developed a complex, community-based IPV intervention to enhance the effectiveness of an existing, proven-effective program of nurse home visitation, the Nurse-Family Partnership, for a subgroup of families in which the woman has experienced recent IPV. A strength of our approach lies in the systematic process used to develop the intervention, in which we drew on multiple perspectives of NFP clients, nurses and community stakeholders, and current theoretical, empirical and practice literature to inform both the content and process of the intervention, and ground it within the context of the NFP and current knowledge related to best practices in nurse home visiting, and in woman- and child-focused IPV intervention delivery. A rigorous approach to conducting qualitative research was implemented thus promoting the overall trustworthiness of the findings upon which the intervention was developed. Data credibility (or internal validity) was strengthened through member checking, data source, data type and researcher triangulation, and reflexivity. Although data were only collected from NFP sites located within the mid-west region of the US, the use of maximum variation sampling provided the opportunity to identify common themes and experiences across a diverse range of sites, nurses and clients.

An interactive process of development allowed for formulation of ideas, dialogue and refinement by a team of researchers with varied disciplinary, practice and research expertise. Although a time intensive process, we contend that it led to a more informed understanding of intervention strategies that could be helpful to women, enhancing the relevance of the intervention for NFP clients and reinforcing its credibility and importance to nurse home visitors who are dealing with heavy caseloads and competing requests for practice changes.

Embedding the IPV intervention within an existing intervention required additional time and resources to ensure theoretical and practical cohesiveness between the original and enhanced intervention. However, the theoretical emphasis within the NFP on enhancing the woman's self-efficacy and parenting capacity, facilitating access to resources, identifying and following the woman's dreams and desires, and seeking small changes over time, are all consistent with the literature on IPV, particularly the emphasis on the uniqueness and complexity of women's experiences of abuse and the need to support women's choices and sense of control [60-62], the importance of women's children in their decisions related to IPV [63,64], and the role of support systems and community services in helping women disengage from an abusive partner [65,66]. Furthermore, we sought to achieve compatibility between the existing NFP guidelines and model elements and the IPV intervention so that the enhanced curriculum would complement the organization's existing values, past experiences and the needs of both clients and nurses [67].

We concluded from the early analysis of the data, that the nursing intervention under development would require both universal elements and those that could be tailored to a woman's specific level of readiness to acknowledge the abuse in her relationship and to promote change within her life. The universal components are framed around safety planning and risk assessment, two interventions that have been shown to positively affect women's use of safety strategies, along with assessment of mental health and substance use patterns, and referral as appropriate. There is evidence that compared to women in the general population, women who are abused have higher prevalence rates of depression [68,69] including post-partum depression [70] and that a majority of women with substance use issues have experienced a history of abuse [68,71]. A key challenge in developing the intervention is that, although women's mental health may be a central resource that helps them address IPV and make changes in their lives [61], enhancing women's mental health is not a core focus of the NFP. For the low-income women served by the NFP, timely access to mental health service is often seriously restricted. Within this context, the nurses' efforts to support women in navigating the system to access mental health care may fall short, not because of lack of interest, but because the system is not responsive to the needs of these women.

With a few exceptions e.g. [20,61], most emerging IPV interventions within health care are time limited and emphasize brief safety planning or empowerment around the time of disclosure. The NFP-IPV intervention has the potential to significantly advance understanding of how nurses can support women who have experienced IPV over time, using a model that draws on the therapeutic relationship and fits with the often long-term process of disengaging from an abusive partner. Even after separation from an abusive partner, many women continue to face long-term safety, health, economic and/or social problems [72,73] that may negatively affect their quality of life and challenge their parenting. The focus of the NFP-IPV on *any *period in the process of change (from a woman being committed to the relationship to separating from an abusive partner) sets it apart from most established or emerging IPV interventions.

The next main step in this research program is to evaluate the effectiveness of the NFP-IPV Intervention in a RCT. An initial step in this process was to conduct, in May 2010, a pilot study to test the feasibility of implementing the NFP IPV intervention and to identify components of the intervention requiring modification. The pilot study was conducted in one of the four original NFP sites that participated in the qualitative case study. The objectives of the pilot study were to: 1) evaluate the acceptability of the training curriculum developed to support nurse home visitors deliver the intervention; 2) refine the procedures for recruiting NFP clients into a RCT; 3) determine the feasibility of integrating the NFP IPV intervention elements into existing NFP curriculum guidelines; 4) confirm that the intervention content and processes were reflective of nurse home visitor practice and of the data collected in Project 1; and 5) evaluate the acceptability of the NFP IPV intervention to nurses and NFP clients.

In general, results of the feasibility study (to be reported in detail elsewhere) indicated that the training curriculum, manuals and tools are effective in educating the nurses about the IPV intervention and that the intervention itself is acceptable to both nurses and clients. Results of the study have been integrated into refinements of the curriculum and clinical protocol, and lessons learned from the research processes have been applied in development and implementation of a RCT, currently underway, to assess the short-term effectiveness of the intervention in improving women's safety and life quality, along with assessing a range of related outcomes, including violence exposure.

## Conclusions

IPV is a complex health and social problem which is shaped by multiple and intersecting personal, social and systems factors; a woman's readiness to address IPV and the options she has available to make changes in her life are also shaped by these factors [74]. Interventions that seek to reduce IPV and its consequences must take the complexity of women's experiences of IPV and of help-seeking into account if they are to be effective. This paper has described an iterative, evidence--and theory-based process for developing a complex intervention to address IPV within the context of an existing intervention for high-risk women and families. The lessons-learned from this process have informed development of a RCT to assess the effectiveness of this IPV intervention, and may also inform development of other interventions to address IPV and family violence more broadly - a documented and pressing need in this field.

## Competing interests

The authors declare that they have no competing interests.

## Authors' contributions

The overall study was conceived by HLM, JC, DLO. SJ designed and coordinated the qualitative case study, participated in data collection and analysis, and led the development of the NFP IPV intervention. MFG participated in data analysis and interpretation and co-led the development of the intervention. DD co-facilitated four focus groups; both DD and DBM participated in the qualitative data analysis. CNW, JC, DLO provided feedback on the emerging intervention. The manuscript was drafted by SJ, MFG, CNW and HM. All authors read and approved the final manuscript.

## Pre-publication history

The pre-publication history for this paper can be accessed here:

http://www.biomedcentral.com/1472-6963/12/50/prepub
