# Erratum to: Development of a nurse home visitation intervention for intimate partner violence

**DOI:** 10.1186/s12913-016-1682-1

**Published:** 2016-08-26

**Authors:** Susan M. Jack, Marilyn Ford-Gilboe, C. Nadine Wathen, Danielle M. Davidov, Diane B. McNaughton, Jeffrey H. Coben, David L. Olds, Harriet L. MacMillan, for NFP IPV Research Team

**Affiliations:** 1 School of Nursing, McMaster University, 1280 Main Street West, Hamilton, ON Canada; 2 Arthur Labatt Family School of Nursing, The University of Western Ontario, London, ON Canada; 3 Faculty of Information and Media Studies, The University of Western Ontario, London, ON Canada; 4 Department of Emergency Medicine, West Virginia University, Morgantown, WV USA; 5 College of Nursing, Rush University, Chicago, IL USA; 6 Departments of Emergency Medicine and Community Medicine, West Virginia University, Morgantown, WV USA; 7 Prevention Research Center for Family and Child Health, University of Colorado Denver, Denver, CO USA; 8 Offord Centre for Child Studies, McMaster University, Hamilton, ON Canada; 9 West Virginia University Injury Control Research Center, Morgantown, WV USA

## Erratum

For full transparency, the authors wish to update the competing interest statement of this article [1] with the following information:

The Prevention Research Center for Family and Child Health (PRC), directed by Dr. David Olds at the University of Colorado School of Medicine, has a contract with the Nurse-Family Partnership© to conduct research to improve the NFP program and its implementation. Dr. David Olds was employed by this center at the time the current study was conducted. The contract with the NFP contributes to the salaries and operations of the PRC. Dr. Olds is the founder of the Nurse-Family Partnership.
